# Mesenchymal Stem Cells: Cell Fate Decision to Osteoblast or Adipocyte and Application in Osteoporosis Treatment

**DOI:** 10.3390/ijms19020360

**Published:** 2018-01-25

**Authors:** Lifang Hu, Chong Yin, Fan Zhao, Arshad Ali, Jianhua Ma, Airong Qian

**Affiliations:** 1 Laboratory for Bone Metabolism, Key Laboratory for Space Biosciences and Biotechnology, School of Life Sciences, Northwestern Polytechnical University, Xi’an 710072, China; hulifang@nwpu.edu.cn (L.H.); yinchong42@mail.nwpu.edu.cn (C.Y.); sofan@mail.nwpu.edu.cn (F.Z.); ArshadAli@mail.nwpu.edu.cn (A.A.); majianhua@mail.nwpu.edu.cn (J.M.); 2 School of Public Health, Lanzhou University, Lanzhou 730000, China

**Keywords:** osteoporosis, mesenchymal stem cell, osteoblast, adipocyte, cell therapy

## Abstract

Osteoporosis is a progressive skeletal disease characterized by decreased bone mass and degraded bone microstructure, which leads to increased bone fragility and risks of bone fracture. Osteoporosis is generally age related and has become a major disease of the world. Uncovering the molecular mechanisms underlying osteoporosis and developing effective prevention and therapy methods has great significance for human health. Mesenchymal stem cells (MSCs) are multipotent cells capable of differentiating into osteoblasts, adipocytes, or chondrocytes, and have become the favorite source of cell-based therapy. Evidence shows that during osteoporosis, a shift of the cell differentiation of MSCs to adipocytes rather than osteoblasts partly contributes to osteoporosis. Thus, uncovering the molecular mechanisms of the osteoblast or adipocyte differentiation of MSCs will provide more understanding of MSCs and perhaps new methods of osteoporosis treatment. The MSCs have been applied to both preclinical and clinical studies in osteoporosis treatment. Here, we review the recent advances in understanding the molecular mechanisms regulating osteoblast differentiation and adipocyte differentiation of MSCs and highlight the therapeutic application studies of MSCs in osteoporosis treatment. This will provide researchers with new insights into the development and treatment of osteoporosis.

## 1. Introduction

Osteoporosis is a systemic bone disease characterized by low bone mass and the deteriorative microarchitecture of bone tissue, which leads to increased bone fragility and the risk of bone fracture [1,2]. As many countries encounter the aging of their populations, osteoporosis has become a significant health concern of the world. In the years 2006 and 2007, it has been reported that osteoporosis affected 200 million women worldwide and caused more than 8.9 million fractures annually, giving rise to an osteoporotic fracture every 3 seconds [3]. At present, more and more people are suffering from osteoporosis. The National Osteoporosis Foundation (NOF) has reported that one in two women and up to one in four men aged 50 and older will break a bone due to osteoporosis. Therefore, osteoporosis has become a major disease of the world and seriously affects people’s health.

Evidence shows that age-related osteoporosis develops in association with reduced bone formation and increased marrow fat accumulation [4,5,6,7]. During osteoporosis development, bone marrow mesenchymal stem cells (MSCs) exhibit reduced capacity to differentiate into osteoblasts and an increased capacity to differentiate into adipocytes, which results in a reduction in bone formation and an increase in marrow fat accumulation [4,8]. It is demonstrated that the shift of cell differentiation of MSCs to adipocytes rather than osteoblasts partly contributes to osteoporosis [8,9]. Therefore, understanding the molecular mechanisms regulating the switch between osteoblast differentiation and adipocyte differentiation of MSCs is important for both understanding osteoporosis occurrence and developing new therapy methods for osteoporosis.

For osteoporosis treatment, the aim is to maintain the normal bone mass. The current osteoporosis therapies are mainly the drug-based agents that inhibit bone resorption or improve bone anabolism. Bisphosphonates, the most widely used agents for osteoporosis treatment, target osteoclast and inhibit bone resorption [10,11,12]. Denosumab, an antibody to the receptor activator of nuclear factor-κB ligand (RANKL), is also one main antiresorptive agent [13,14,15]. There are a few bone anabolic agents. Teriparatide, a parathyroid hormone (PTH) analog, is an anabolic agent that functions by inducing bone formation [16]. In addition, romosozumab, an antibody to sclerostin, is a new anabolic agent that both enhances bone formation and inhibits bone resorption [17,18]. However, most antiresorptive agents showed side effects during the treatment of osteoporosis [19,20] and there are fewer bone anabolic agents. Therefore, there is an urgent need to develop new treatments for osteoporosis. With the rapid development of cell-based therapy, MSCs have become the focus of new treatments of osteoporosis.

Here, we review the current understanding of MSCs and summarize the recent advances of the molecular mechanisms regulating osteoblast and adipocyte differentiation of MSCs. Given the special role of MSCs in osteoporosis and their multipotency, MSCs-based preclinical and clinical studies for osteoporosis are introduced by focusing on bone marrow MSCs (BM-MSCs) and adipose tissue-derived MSCs (AD-MSCs). This review will provide researchers an increase understanding of MSCs and give guidance for the future research on osteoporosis treatment.

## 2. Mesenchymal Stem Cells (MSCs) and Bone Marrow MSCs (BM-MSCs) in Osteoporosis Development

MSCs, also known as marrow stromal cells, are multipotent adult stem cells that exist in almost all post-natal organs and tissues, such as bone marrow, fat tissues, liver, muscle, and the umbilical cord [21,22]. MSCs were first discovered in bone marrow by Friedenstein et al. [23]. They found the non-hematopoietic stem cell population in bone marrow and observed that the cells were adherent, spindle-shaped, and able to form single-cell-derived colonies [23]. Further, Friedenstein et al. reported that the non-hematopoietic stem cells showed stem-like characteristics [24]. Thereafter, MSCs were isolated from other tissues, including adipose [25,26], umbilical cord [27], muscle [28], and dental pulp [29]. These demonstrate the ubiquitous distribution of MSCs.

**Figure 1:**
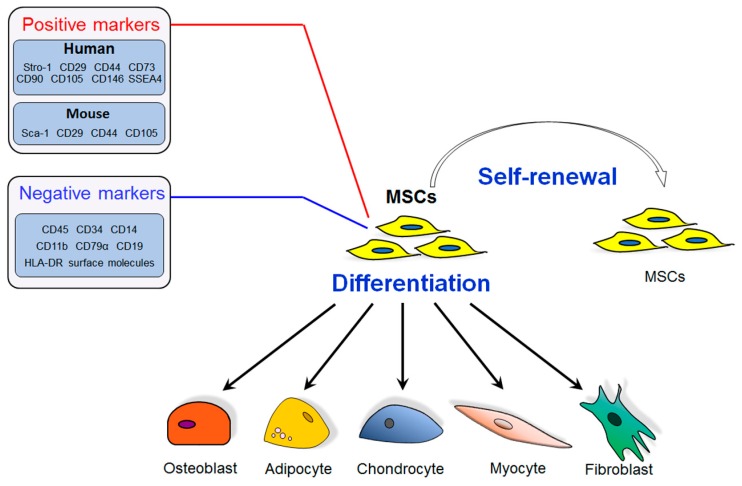
Schematic diagram of the characteristics of mesenchymal stem cells (MSCs). There are both positive markers and negative markers for identifying MSCs. MSCs possess the characteristics of self-renewing and differentiating into multiple cell types, including osteoblast, adipocyte, chondrocyte, myocyte, and fibroblast.

MSCs are capable of self-renewing and differentiating into multiple cell lineages, including osteoblasts, adipocytes, and chondrocytes [30,31] (Figure 1). To define MSCs in a common standard, the Mesenchymal and Tissue Stem Cell Committee of the International Society for Cellular Therapy has proposed the minimal criteria to define human MSCs [32]. First, MSCs must be plastic-adherent when maintained in standard culture conditions. Second, MSCs must express CD105, CD73, and CD90, and lack expression of CD45, CD34, CD14, CD11b, CD79α, CD19, and human leukocyte antigen-antigen D related (HLA-DR) surface molecules. Third, MSCs must differentiate to osteoblasts, adipocytes, and chondroblasts in vitro. As there are no specific markers for MSCs, both positive markers and negative markers are applied to identify MSCs. The positive markers are expressed by MSCs, while negative markers should not expressed by MSCs. Accordingly, in humans, Stro-1, CD29, CD44, CD73, CD90, CD105, CD146, and SSEA4 are positive combination markers for MSCs [33,34], while in mice, Sca-1, CD29, CD44, and CD105 are the positive markers [34]. Additionally, there are negative markers that distinguish MSCs from hematopoietic or endothelial cells, including CD45, CD34, CD14, CD11b, CD79α, CD19, and HLA-DR surface molecules [32] (Figure 1).

BM-MSCs are MSCs residing in bone marrow and have multiple differentiation potentials. BM-MSCs can differentiate into osteoblasts and adipocytes, which play an important role in maintaining normal bone stability. It has been demonstrated that the altered proliferation and differentiation of BM-MSCs is one main cause of osteoporosis [35,36]. Moerman et al. have reported that in old mice, the adipocytes are increased and osteoblasts are decreased, accompanied by reduced bone mineral density (BMD) [4]. Further study on the differentiation potential of MSCs isolated from old (26-month-old) mice showed a high ability to differentiate into adipocytes rather than osteoblasts [4]. Shen et al. indicated an inverse relationship between pelvic bone marrow adipose tissue and BMD, suggesting that low BMD may be due to the shift in preferential differentiation of MSCs from osteoblasts to adipocytes [7]. These studies demonstrate that a shift in the fate of MSCs (more adipocytes, less osteoblasts) contributes to osteoporosis.

## 3. The Molecular Mechanisms Regulating Osteoblast and Adipocyte Differentiation of MSCs

MSCs are capable of differentiating into osteoblasts and adipocytes. The specific differentiation direction is precisely regulated by biological, physical, and chemical factors. Changes in these factors will result in different outcomes for MSCs. As the shift in cell differentiation of MSCs to adipocytes rather than osteoblasts contributes to osteoporosis, here we summarize the advances in the understanding of the molecular mechanisms regulating osteoblast (osteogenic) and adipocyte (adipogenic) differentiation of MSCs. We mainly focus on the intracellular biological factors, including transcription factors, signaling pathways, and microRNAs. In addition, the external physical and chemical factors including mechanical stimuli, radiation, and high fat diet are also introduced.

### 3.1. Transcription Factors

Transcription factors play essential roles in regulating the differentiation of MSCs into osteoblasts or adipocytes. Transcription factors initiate and promote the specific-cell type differentiation process by upregulating the expression of genes responsible for the specific-cell type.

Runt-related transcription factor 2 (runx2) and osterix are two key transcription factors that promote osteoblast differentiation of MSCs [37]. Runx2 is also known as core binding factor α1 (Cbfa1). Runx2 deficiency results in a complete lack of bone formation due to maturational arrest of osteoblasts, showing the necessity of runx2 in osteogenesis [38]. Runx2 promotes osteoblast differentiation but inhibits adipocyte differentiation of MSCs [39]. Runx2-deficient cells fail to differentiate into mature osteoblasts but appear as adipocyte phenotypes [40]. Runx2 functions as an osteogenic regulator by forming a heterodimer with cotranscription factor core binding factor β (Cbfβ) and binding to DNA [40,41]. As the key transcription factor for osteoblast differentiation, runx2 is a target of many signaling pathways [42]. Besides runx2, osterix is another essential transcription factor for osteogenic differentiation of MSCs. Osterix is also called Sp7 and belongs to the Sp transcription factor family. Osterix is demonstrated to function at the downstream of runx2 and can be activated by runx2 [43,44,45]. In osterix-null mice, the MSCs cannot differentiate into osteoblasts and no bone formation occurs [43]. Besides runx2 and osterix, other transcription factors (TAZ, forkhead box C2 (Foxc2), and the proteins Twist-1 and Twist-2) also regulate osteogenic differentiation of MSCs [46,47,48] and twist proteins suppress osteogenic differentiation of MSCs.

For directing MSCs differentiation into adipocytes, peroxisome proliferation-activated receptor γ (PPARγ) is the most important and widely studied transcription factor [49]. PPARγ plays a vital role in promoting adipogenic differentiation of MSCs by regulating the expression of adipogenic genes. PPARγ shows proadipocytic and antiosteoblastic effects [49]. Inhibition of PPARγ suppresses adipogenesis [50]. Additionally, CCAAT/enhancer binding protein α (C/EBPα) is a key transcription factor for adipogenesis. In 1994, Lin et al. reported that C/EBPα was sufficient to trigger the adipogenic differentiation of preadipocytes [51]. Moreover, some transcription factors have been reported to play suppressive roles in regulating adipogenic differentiation of MSCs, including GATA-2, forkhead transcription factor 1 (Foxa1), and Homeobox C8 (HOXC8). Inhibition or knockdown of these transcription factors promotes adipogenic differentiation, while activation or overexpression of them suppresses the adipogenic differentiation of MSCs [52,53]. These findings suggest that the adipogenic differentiation of MSCs have been precisely regulated by numerous transcription factors, including both positive and negative regulators.

Although numerous transcription factors have been identified for regulating osteoblast or adipocyte differentiation of MSCs, further research for screening out transcription factors playing dual roles in regulating both osteoblast and adipocyte differentiation of MSCs is necessary. The underlying mechanisms of these transcription factors regulating the differentiation of MSCs need further study.

### 3.2. Signaling Pathways

Over the recent decades of intensive studies, several signaling pathways have been demonstrated to regulate osteoblast and adipocyte differentiation of MSCs. The bone morphogenic protein (BMP) signaling and wingless and int-1 (Wnt) signaling are two critical signaling pathways.

BMPs are members of the transforming growth factor-β (TGF-β) superfamily and play important roles in regulating bone formation [54]. There are over 30 different BMPs that function through either canonical BMP signaling or non-canonical BMP signaling. The canonical BMP signaling is Smad-dependent while the non-canonical BMP signaling does not depend on Smad. Through binding to bone morphogenetic protein receptor I (BMPR-I) and BMPR-II, BMPs activate the canonical BMP signaling (BMP ligands, receptors, and Smads) or the non-canonical signaling (p38 mitogen-activated protein kinase (MAPK) pathway) [54]. The canonical BMP signaling induces phosphorylation of Smad1/5/8, which form complexes with Smad4 and translocate into the nucleus to regulate target gene expression. The non-canonical BMP signaling functions mainly through transforming growth factor-β activated kinase 1 (TAK1) [54]. It has been demonstrated that both the canonical BMP signaling and non-canonical BMP signaling are important in determining MSCs differentiation. BMP2, BMP4, BMP6, BMP7, and BMP9 have shown dual roles in effectively inducing both osteogenic differentiation and adipogenic differentiation of MSCs in vitro and in vivo [55]. BMP2 is critical for osteogenic differentiation of MSCs by targeting runx2/Cbfa1 [56,57] and shows dose-dependent effects. High concentration of BMP2 accelerates osteoblast differentiation while low concentration of BMP2 promotes adipocyte formation in the C3H10T1/2 mesenchymal cell line [58]. BMP4 is capable of triggering adipocyte lineage commitment of C3H10T1/2 cells [59]. Both BMP induced canonical Smad1/5/8 signaling and non-canonical MAPK signaling promote adipogenesis by activating PPARγ [60]. These studies indicate the important dual roles of BMP signaling in regulating MSCs differentiation. However, given the diversity of BMPs and the dose-dependent effects, the detailed mechanisms need to be deeply investigated.

Wnt signaling is essential in cell fate determination, proliferation, and differentiation. The Wnt family is composed of multiple secreted glycoproteins that are post-translationally modified by the addition of lipid. Like BMP signaling pathways, there is also canonical Wnt signaling and non-canonical Wnt signaling. The best-studied Wnt signaling is the canonical Wnt signaling. It is also called Wnt/β-catenin signaling for its dependency on β-catenin. Through binding to one 7 transmembrane spanning protein frizzled (Frz) receptor and the coreceptor low-density lipoprotein receptor-related protein 5/6 (LRP5/6), Wnt/β-catenin signaling is activated, thus preventing the phosphorylation and degradation of β-catenin [61]. The unphosphorylated β-catenin subsequently translocate into the nucleus to determine the differentiation fate of MSCs [62]. Increasing evidence demonstrates that Wnt/β-catenin signaling shows proosteoblastic and antiadipocytic differentiation effects [63,64]. Wnt/β-catenin signaling is critical in promoting osteoblastic differentiation and maintaining bone mass [65,66]. Byun et al. has reported that Wnt3a stimulates osteogenic differentiation via activating TAZ [67]. By studying FABP4-Wnt10b mice that overexpress Wnt10b in marrow, Bennett et al. [68] found that overexpression of Wnt10b increased bone mass and strength and prevented the aging-related bone loss. The expression of Wnt10b in mesenchymal progenitors promoted osteoblastogenesis by inducing the expression of *runx2*, *distal-less homeobox 5* (*Dlx5*), and *osterix*, and suppressed adipogenesis by inhibiting C/EBPα and PPARγ [68]. The similar results were also obtained by Kang et al. [69]. Furthermore, Wnt6 and Wnt10a also showed a promotion effect on osteogenesis but an inhibitory effect on adipogenesis in a β-catenin-dependent way [70]. Thus, the studies show that inhibition of Wnt/β-catenin signaling suppressed osteogenesis but promoted adipogenesis [71]. All these strongly indicate the importance of Wnt signaling in regulating the osteogenic and adipogenic differentiation of MSCs.

**Figure 2:**
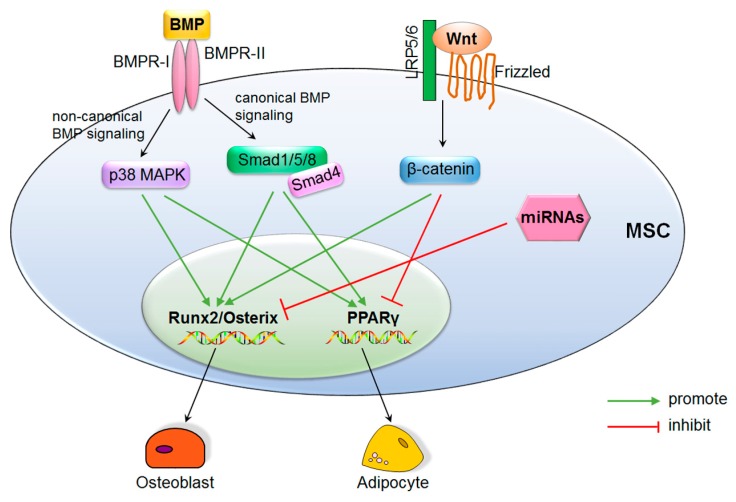
Integration of bone morphogenic protein (BMP) and wingless and int-1 (Wnt) signaling pathways, miRNAs, and key transcription factors in regulating osteoblast and adipocyte differentiation of MSCs. The BMP signaling, Wnt signaling, and miRNAs regulate osteoblast differentiation or adipocyte differentiation of MSCs by targeting key transcription factors such as runx2, osterix, or PPARγ.

From the BMP and Wnt signaling studies, we can see the dual roles of these signaling pathways in regulating bidirectional differentiation of MSCs (Figure 2). As there are multiple signaling pathways involved in MSCs differentiation, crosstalk may exist among these pathways. Further studies for constructing the network of these pathways are needed to provide more evidence illustrating the mechanisms regulating osteogenic and adipogenic differentiation of MSCs.

### 3.3. MicroRNAs

MicroRNAs (miRNAs) are noncoding, ~22 nucleotide RNAs. Since the discovery of the first miRNA [72], a rush of miRNA related research has been rapidly undertaken. More recently, the role of miRNAs in the lineage commitment of MSCs has attracted more and more attention. Multiple miRNAs have been demonstrated as regulators of MSCs differentiation and to play key roles in bone [73,74,75,76]. miR-21 promotes osteogenic differentiation of MSCs derived from human umbilical cord by activating the phosphoinositide 3-kinase (PI3K)-AKT-glycogen synthase kinase 3 β (GSK3β) pathway, by inducing β-catenin accumulation and runx2 activation [77]. However, a contrary result was obtained by Kim et al., showing that miR-21 enhanced adipogenic differentiation via modulating the TGF-β signaling in human adipose tissue-derived MSCs (AD-MSCs) [78]. These contrary results may be due to the different cell type and suggest that the same miRNA may play different roles in different MSCs. To date, most miRNAs show antiosteoblastic differentiation but proadipocytic differentiation effects in MSCs. miR-138 is a negative regulator of osteogenic differentiation of hMSC in vitro and in vivo [79]. miR-204 shows proadipocytic and antiosteoblastic differentiation effects on MSCs by inhibiting runx2 expression [80], while miR-637 shows similar effects by directly suppressing osterix expression [81]. miR-31 also displays inhibitory effect on osterix during osteogenic differentiation of MSCs [82]. All these studies demonstrate that most miRNAs promote adipogenic differentiation but suppress osteogenic differentiation by inhibiting runx2 or osterix expression (Figure 2), suggesting their importance for the cell fate decision of MSCs. However, the role of miRNAs in regulating MSCs differentiation has only recently been revealed and considerable work needs to be done to discover more miRNAs and uncover their function mechanisms.

All these findings demonstrate that the key transcription factors, such as runx2, osterix, and PPARγ, are the targets of the signaling pathways and miRNAs in regulating osteoblast or adipocyte differentiation of MSCs (Figure 2). Moreover, these signaling pathways or miRNAs may function by forming a network (Figure 2).

### 3.4. Other Factors

The above demonstrates the critical role of intracellular factors (e.g., transcription factors, signaling pathways, miRNAs) in regulating osteoblast or adipocyte differentiation of MSCs with the transcription factors playing a decisive role. Besides this, reports from others and our laboratory have demonstrated that external factors, such as mechanical stimuli, radiation, and high fat diet, also play important roles in regulating the differentiation fate of MSCs.

MSCs have been shown to be mechanosensitive and their differentiation fate can be regulated by mechanical stimuli [83,84]. Numerous studies have confirmed that the osteoblast or adipocyte differentiation of MSCs is obviously regulated by various mechanical factors, such as exercise, vibration, and microgravity. Studies illustrate that exercise promotes osteoblast differentiation and inhibits adipocyte differentiation of MSCs [85,86]. Menuki et al. reported that 28 days of climbing exercise significantly increased bone volume and osteoblast number, while decreasing marrow adipocyte volume and number in 8-week-old C57BL/6J male mice [85]. They further found that climbing exercise promoted osteoblast differentiation and inhibited terminal adipocyte differentiation of bone marrow stromal cells. Similar findings have been obtained in 4-week-old C57BL/6 male mice by Maredziak et al. [86]. Further, vibration, as a surrogate for exercise, has also shown a key role in regulating osteoblast or adipocyte differentiation of MSCs. In vivo studies demonstrate that vibration with low magnitude high frequency (LMHF) promotes osteoblast differentiation while inhibits adipocyte differentiation of MSCs [87,88]. In vitro studies have also been conducted to illustrate the effect of vibration on MSCs differentiation and the underlying molecular mechanism. Ozcivici’s group found that 1-week treatment (15 min/day) of LMHF (0.15 g, 90 Hz) vibration promoted osteoblastic differentiation and suppressed adipogenic differentiation of mouse BM-MSCs, showing a significant increase of *runx2* and reduction of *PPARγ* and *C/EBPα* [89,90]. Chen et al. also reported that 0.3 g acoustic vibration at 800 Hz (30 min/day) promoted osteogenic differentiation and suppressed adipogenic differentiation via upregulating *runx2* expression and downregulating *PPARγ* [91]. In addition, Zhou et al. showed that LMHF (0.3 g, 40 Hz, 30 min/12 h) vibration promoted osteogenic differentiation of rat BM-MSCs through activating extracellular signal-regulated kinase 1/2 (ERK1/2) signaling and upregulating runx2 expression [92]. As the ERK1/2 signaling pathway regulates mechanotransduction [93] and is important for phosphorylation and activation of runx2 [94,95], the LMHF vibration may promote osteoblast differentiation of MSCs via ERK1/2 signaling. While most studies show proosteoblastic and antiadipocytic differentiation effects on MSCs [96,97], some contrary findings are reported. You’s group and Yu’s group found that LMHF vibration inhibited osteoblastic differentiation but promoted adipogenic differentiation of rat BM-MSCs [98,99]. You’s group reported that LMHF (0.3 g, 60 Hz, 1 h/1 day) vibration decreased osterix expression and inhibited mineralization in MSCs [98], while Yu’s group found that LMHF (0.3 g, 40 Hz, 15 min/day) vibration significantly increased the expression of PPARγ, *C/EBPα*, *adiponectin*, and the oil droplets [99]. Yu’s study also demonstrated that LMHF vibration promoted adipogenic differentiation of MSCs through activating p38 MAPK signaling. The above inconsistent findings from vibration studies may be due to different vibration parameters (e.g., frequency, magnitude) and experimental design (e.g., treatment time, culture condition). Moreover, other types of mechanical stimuli, such as stretch/tensile strain [100,101,102,103], pressure/compressive stress [104,105], and shear stress [106,107], have also been studied in regulating MSCs differentiation and most show proosteoblastic and antiadipocytic differentiation roles. Although there are few contrary results, the above findings demonstrate that the mechanical loading promotes osteoblast differentiation and suppresses adipocyte differentiation of MSCs and may through activating ERK1/2, p38 MAPK signaling [92,99,101,103,104], or others. The above findings demonstrate the importance of mechanical stimuli (loading) on favoring osteoblast differentiation of MSCs. Moreover, the effect of microgravity/weightlessness (mechanical unloading) on MSCs differentiation also confirms the importance of mechanical stimuli on MSCs differentiation by showing proadipocytic and antiosteoblastic effects on MSCs [108,109]. Studies from others and our laboratory showed that simulated microgravity inhibited osteogenic differentiation and promoted adipogenic differentiation of MSCs both in vivo and in vitro [110,111,112,113,114]. The possible molecular mechanism is that decreased integrin/mitogen-activated protein kinase (MAPK) signaling [115], suppression of RhoA activity [116], decreased ERK1/2 phosphorylation [112,113], and increased p38 phosphorylation [112] result in downregulation of runx2 and upregulation of PPARγ [117]. Other unknown pathway may exist and need further study.

Besides mechanical stimuli, other external physical or chemical factors, such as radiation and high fat diet, also play roles in regulating MSCs differentiation [118,119,120]. Islam et al. studied the effects of radiation (0–16 Gy) on both human pluripotent stem cells and BM-MSCs [118]. They found that osteogenic differentiation was reduced in both MSCs while adipogenic differentiation was increased in human pluripotent stem cells by radiation, and the downregulation of Wnt signaling may contribute to determine the cell fate of irradiated MSCs. As adipocytes and osteoblasts originate from the same progenitor cells (MSCs), a correlation between fat and bone attracted attention [121]. It has been demonstrated that high fat diet inhibits bone formation by blocking differentiation of osteoblast progenitor cells and promotes adipogenesis of MSCs by regulating osteoblastic or adipocytic genes (e.g., *PPARγ*) [119,120].

Taken together, external physical or chemical factors are critical in regulating osteoblast and adipocyte differentiation of MSCs through modulating the intracellular signaling pathways and key transcription factors.

## 4. Therapeutic Applications of MSCs for Osteoporosis

MSCs have the capacity to self-renew and to differentiate into various cell types. Thus, they have become an ideal cell source for cell-based therapy for a variety of diseases and have shown therapeutic effects in both animal models and clinical trials [122,123].

For osteoporosis, the altered differentiation potential of BM-MSCs that favor adipocytes rather than osteoblasts is one main cause. Therefore, treatment strategy aimed at altering the differentiation direction of MSCs or augmenting normal endogenous MSCs could be a potential method for osteoporosis therapy. Thus, MSCs cell-based therapies have attracted considerable attention for treating osteoporosis [124]. Here we just focus on the therapeutic applications of two sources of MSCs, BM-MSCs and AD-MSCs, for osteoporosis treatment in both preclinical studies and clinical trials.

### 4.1. Bone Marrow MSCs (BM-MSCs)

The most commonly used MSCs are the BM-MSCs, which have been broadly studied in bone regeneration and bone repair due to their easy accessibility and high osteogenic differentiation capacity. Studies in animal models demonstrate that both autologous and allogeneic BM-MSCs are applicable in osteoporosis treatment by cell transplantation, which are either local or systemic. Wang and colleagues locally transplanted the autologous BM-MSCs into the distal femurs in an ovariectomy (OVX)-induced osteoporosis rabbit model [125]. They found more bone apposition, increased trabecular thickness, improved microstructures with newly formed osteoids, enhanced trabecular thickness, and stronger stiffness of bone in the MSC-alginate treatment group after eight weeks [125]. Ichioka et al. locally injected the normal allogeneic BM-MSCs into the bone marrow cavity of senescence accelerated mouse prone 6 (SAMP6) mice (a substrain of senescence-accelerated mice, an osteoporosis mouse model) and found that BM-MSCs increased trabecular bone, attenuated the loss of BMD, and prevented osteoporosis [126]. A similar finding was obtained by Ocarino et al. [127]. They constructed an OVX-induced osteoporosis rat model and conducted intra-bone marrow injection of allogeneic BM-MSCs that were isolated from healthy rats. The results showed that the allogeneic BM-MSCs improved the femur bone mass of osteoporotic rats [127]. More recently, the systemic infusion of allogeneic BM-MSCs has been demonstrated to prevent the reduction of bone mass and strength in the glucocorticoid-induced osteoporosis mouse model [128]. The donor BM-MSCs inhabit and function in recipient bone marrow, thus promoting osteoblastogenesis and in turn maintaining bone formation [128]. Furthermore, Kiernan et al. revealed that systemic injection of allogeneic MSCs markedly increased bone formation and sustained microarchitectural competence in an age-related osteoporosis mouse model, suggesting the application potential of MSCs in human aged-related osteoporosis therapy [129]. These studies demonstrate that BM-MSCs are effective for treating osteoporosis in animal models (Table 1), suggesting their clinical application potential for osteoporosis.

Recently, there are beginning clinical trials of BM-MSCs for osteoporosis treatment. One clinical trial sponsored by Red de Terapia Celular has been conducted at Hospital Clinico Virgen de la Arrixaca in Spain. In this clinical trial, autologous BM-MSCs were collected approximately 30 days before infusion. Then, mononuclear bone marrow cells were separated and cultured in good manufacturing practices (GMP) conditions to purify and obtain MSCs in the established dose range. The BM-MSCs were fucosylated and intravenous injected into osteoporosis patients. This clinical trial is in Phase I and still in the process of recruiting. It is estimated to be completed in December 2018 (Available online: http://clinicaltrials.gov, NCT02566655, Table 2).

### 4.2. Adipose Tissue-Derived MSCs (AD-MSCs)

Recently, AD-MSCs have become one popular cell source of MSCs due to their easy isolation, greater abundance, and high production [130]. It has been demonstrated that the proliferation and osteogenic differentiation of AD-MSCs is less affected by age and multiple passage, making AD-MSCs a potential source for cell-based therapy [131]. Cho et al. found that human adipose tissue-derived stromal cell (ADSC) therapy prevented OVX-induced bone loss [132]. Mirsaidi et al. have also evaluated the use of AD-MSCs as a treatment strategy for age-related osteoporosis in a SAMP6 osteoporotic mouse model [133]. They found that a single intratibial injection of isogeneic AD-MSCs significantly improved trabecular bone quality and induced a significant increase in several molecular markers of bone turnover [133]. In addition, AD-MSCs also increased bone mineral density and new bone formation in an OVX-induced osteoporotic rabbit model [134]. All these demonstrate that AD-MSCs transplantation is an effective cell therapy method for osteoporosis treatment.

One clinical trial in Phase II using human AD-MSCs for treatment of proximal humeral fracture as a model for osteoporotic fracture has been conducted by University Hospital in Basel, Switzerland. (Available online: http://clinicaltrials.gov, NCT01532076). The AD-MSCs were isolated from the patient, seeded within a composite graft, and transplanted back into the fracture site. The clinical/radiological follow-up and functional assessment were performed. However, the trial was terminated and no results were reported (Table 2).

The preclinical studies in animal models have demonstrated that BM-MSCs and AD-MSCs are effective in treating osteoporosis (Table 1). The clinical trials of BM-MSCs and AD-MSCs in osteoporosis treatment have also been conducted (Table 2), but they have only recently begun and no results have been reported at present. Therefore, more clinical trials with a greater number of patients are needed to determine the therapeutic effects of BM-MSCs and AD-MSCs for treating osteoporosis.

## 5. Concerns on the Clinical Application of MSCs in Osteoporosis Treatment and Future Direction

Although the MSCs have been applied for osteoporosis treatment in both preclinical and clinical studies and showed therapeutic effects, some concerns on the clinical application still exist. Firstly, the bone marrow homing efficiency of MSCs after systemic transplantation. Secondly, the long-term survival and the uncertainty of MSCs fate following cell transplantation.

Aiming at the above concerns, studies have been conducted to solve these problems. To improve bone marrow homing efficiency of transplanted MSCs, researchers modify MSCs using genetic methods. Genetic modification of MSCs with C-X-C chemokine receptor type 4 (CXCR4), a chemokine receptor largely responsible for stromal-derived factor-1 (SDF-1)-mediated bone marrow homing, increases bone marrow homing of transplanted MSCs and restores bone mass and bone formation in mice with glucocorticoid-induced osteoporosis [135]. In addition, the ectopic expression of α4 integrin on MSCs greatly increases bone homing of MSCs and the engrafted MSCs form osteoblasts and osteocytes in an immunocompetent mouse model [136]. The genetic methods have also been applied to ensure the long-term survival and maintain differentiation capacity of MSCs following cell transplantation. Combination of p53 knockdown and human telomerase reverse transcriptase (hTERT) overexpression increases life span of MSCs and retains cell differentiation potential [137]. Overexpression of basic fibroblast growth factor (bFGF) or platelet-derived growth factor B (PDGF-BB) in MSCs promotes cell proliferation and osteogenesis by activating ERK1/2 signaling pathway, and inhibits adipogenesis by downregulating PPARγ [138]. Moreover, genetic modification of MSCs with hepatocyte growth factor (HGF) promotes the expression of osteogenic genes (*runx2*, *ALP* (*alkaline phosphatase*), *OC* (*osteocalcin*)) of MSCs and prevents bone loss in OVX-induced osteoporotic mice [139]. The study also suggests that transplanted MSCs can act in paracrine manner to prevent bone loss [139]. Besides genetic modification of MSCs within cells, researchers also try to improve in vitro MSCs culture system to obtain high-quality MSCs. One approach is to adjust the culture conditions before cell transplantation. Hypoxic culture has been demonstrated to promote cell proliferation, enhance cell differentiation potential, and increase cell homing of MSCs [140].

The above studies indicate that modification of MSCs either within cell (genetic modification) or outside the cell (adjusting external factor) can improve MSCs properties. Therefore, based on the understanding of MSCs properties and the molecular mechanisms regulating osteoblast and adipocyte differentiation of MSCs, researchers will obtain desired MSCs through modifying MSCs by combining both intracellular and extracellular factors. This will be the future direction for both preclinical and clinical studies, making the MSCs-based cell therapy safer and more effective for clinical application for osteoporosis.

## 6. Conclusions and Perspectives

With the aging population increases in the world, osteoporosis has become a significant health concern. Although there are some drug-based agents for osteoporosis treatment, some side effects exist. Therefore, alternative treatments are urgently required. It has been demonstrated that the shift of cell differentiation of MSCs to adipocytes rather than osteoblasts contributes to osteoporosis. MSCs, with their multipotency, have become the focus of cell therapy. Thus, treatment strategy aimed at altering the differentiation direction of MSCs (promoting osteoblast differentiation and inhibiting adipocyte differentiation) could be a potential method for osteoporosis therapy.

For regulating the osteoblast or adipocyte differentiation of MSCs, intracellular biological factors, including transcription factors, signaling pathways, and miRNAs, show important roles. Runx2 and osterix are two critical osteogenic transcription factors, while PPARγ is the adipocyte-specific transcription factor. The activation of these transcription factors in MSCs leads to the specific cell lineage commitment. BMP signaling and Wnt signaling show dual roles in regulating osteoblast and adipocyte differentiation of MSCs by targeting the downstream transcription factors runx2, osterix, or PPARγ. In addition, miRNAs, one type of newly discovered regulators, show a suppressive effect on osteogenic differentiation but promotive effect on the adipogenic differentiation of MSCs. Moreover, external physical and chemical factors, such as mechanical stimuli, radiation, and high fat diet, are important in regulating the osteoblast or adipocyte differentiation of MSCs. Mechanical loading promotes osteoblast differentiation and suppresses adipocyte differentiation of MSCs through regulating intracellular signaling pathways and transcription factors. The radiation and high fat diet both show antiosteoblastic and proadipocytic differentiation effects on MSCs. These findings provide more understanding of the molecular mechanisms regulating MSCs differentiation and may provide potential targets and new methods for manipulating the MSCs to alter their cell fate.

MSCs-based preclinical studies in animal models show that both BM-MSCs and AD-MSCs are effective in osteoporosis treatment. It has been demonstrated that both autologous and allogeneic MSCs are applicable in osteoporosis treatment by either local or systemic treatment. All these findings strongly suggest a great clinical application potential of MSCs for osteoporosis. However, the clinical trials of MSCs in osteoporosis treatment have just begun and no results have been reported at present. Further clinical trials need to be done but there are still some concerns need to be considered. The bone marrow homing efficiency of MSCs, the long-term survival and the uncertainty of MSCs’ fate after cell transplantation are the main concerns on the clinical application of MSCs for osteoporosis. Aiming at these concerns, many ongoing studies have indicated that modification of MSCs either within-cell (genetic modification) or outside the cell (adjusting external factor) could improve MSCs properties. These studies demonstrate the possible future direction for both preclinical and clinical studies of MSCs in osteoporosis treatment.

In summary, osteoporosis is partly due to the shift of cell differentiation of MSCs to adipocytes rather than osteoblasts. MSCs-based cell therapy is applicable in osteoporosis treatment. Based on the understanding of MSCs properties and the molecular mechanisms regulating osteoblast and adipocyte differentiation of MSCs, modification of MSCs will make the MSCs-based cell therapy safer and more effective for clinical application for osteoporosis in the future.

## Figures and Tables

**Table 1:** Preclinical studies using BM-MSCs and AD-MSCs in the osteoporosis treatment in animal models.

Cell Type	Treatment Method	Therapeutic Outcomes	References
BM-MSCs	Local transplantation of autologous BM-MSCs	Increased trabecular thickness, improved microstructures with newly formed osteoids, and enhanced trabecular thickness and stiffness of bone.	[125]
BM-MSCs	Local injection of the normal allogeneic BM-MSCs	Increased trabecular bone, attenuated the loss of BMD, improved the femur bone mass and prevented osteoporosis.	[126,127]
BM-MSCs	Systemic injection of allogeneic BM-MSCs	Promoted osteoblastogenesis, maintained bone formation, and prevented the reduction of bone mass and strength in osteoporotic mouse model. Increased bone formation and sustained microarchitectural competence in a mouse model of age-related osteoporosis.	[128,129]
AD-MSCs	Systemic injection of allogeneic AD-MSCs	Prevented OVX-induced bone loss.	[132]
AD-MSCs	Local injection of autologous AD-MSCs	Improved trabecular bone quality and induced a significant increase in several molecular markers of bone turnover. Promoted osteogenesis, inhibited adipogenesis, and increased bone mineral density and new bone formation.	[133,134]

BM-MSCs: bone marrow mesenchymal stem cells; BMD: bone mineral density; AD-MSCs: adipose tissue-derived mesenchymal stem cells; OVX: ovariectomy.

**Table 2:** Clinical trials with BM-MSCs and AD-MSCs in osteoporosis treatment.

Cell Type	Treatment Method	Disease Treated	No. of Patients	Dose (No. of Treatment)	Phase	Therapeutic Outcomes	Clinical Trial No.
BM-MSCs	Intravenous injection of autologous BM-MSCs that were fucosylated	Osteoporosis, Spinal fractures	10	First 4 patients receive 2 million cells/kg; last 6 receive 5 million cells/kg (Single)	Phase I	Still in progress, no results	NCT02566655
AD-MSCs	AD-MSCs were seeded within a composite graft and transplanted back into the fracture site	Osteoporotic fractures	8	Unknown	Phase II	Terminated, no results	NCT01532076

## Abbreviations

AD-MSCs	adipose tissue-derived MSCs
ADSC	adipose tissue-derived stromal cell
ALP	alkaline phosphatase
bFGF	basic fibroblast growth factor
BMD	bone mineral density
BM-MSCs	bone marrow MSCs
BMP	bone morphogenic protein
BMPR	bone morphogenetic protein receptor
Cbfa1	core binding factor α1
Cbfβ	core binding factor β
C/EBPα	CCAAT/enhancer binding protein α
CXCR4	C-X-C chemokine receptor type 4
Dlx5	distal-less homeobox 5
ERK1/2	extracellular signal-regulated kinase 1/2
Foxa1	forkhead transcription factor 1
Foxc2	forkhead box C2
Frz	frizzled
GMP	good manufacturing practices
GSK3β	glycogen synthase kinase 3 β
HGF	hepatocyte growth factor
HLA-DR	human leukocyte antigen-antigen D related
HOXC8	homeobox C8
hTERT	human telomerase reverse transcriptase
LMHF	low magnitude high frequency
LRP5/6	low-density lipoprotein receptor-related protein 5/6
MAPK	mitogen-activated protein kinase
miRNA	microRNA
MSCs	mesenchymal stem cells
OC	osteocalcin
OVX	ovariectomy
PDGF-BB	platelet derived growth factor B
PI3K	phosphoinositide 3-kinase
PPARγ	peroxisome proliferation-activated receptor γ
PTH	parathyroid hormone
RANKL	receptor activator of nuclear factor-kappa B ligand
Runx2	runt-related transcription factor 2
SAMP6	senescence accelerated mouse prone 6
SDF-1	stromal-derived factor-1
TAK1	transforming growth factor-β activated kinase 1
TGF-β	transforming growth factor-β
Wnt	wingless and int-1
